# Experimental Identification of Electric Field Excitation Mechanisms in a Structural Transition of Tokamak Plasmas

**DOI:** 10.1038/srep30720

**Published:** 2016-08-04

**Authors:** T. Kobayashi, K. Itoh, T. Ido, K. Kamiya, S.-I. Itoh, Y. Miura, Y. Nagashima, A. Fujisawa, S. Inagaki, K. Ida, K. Hoshino

**Affiliations:** 1 National Institute for Fusion Science, Toki 509-5292, Japan; 2 Research Center for Plasma Turbulence, Kyushu University, Kasuga 816-8580, Japan; 3 National Institutes for Quantum and Radiological Science and Technology, Naka 311-0193, Japan; 4 Research Institute for Applied Mechanics, Kyushu University, Kasuga 816-8580, Japan; 5 Japan Atomic Energy Agency, Tokai 319-1184, Japan

## Abstract

Self-regulation between structure and turbulence, which is a fundamental process in the complex system, has been widely regarded as one of the central issues in modern physics. A typical example of that in magnetically confined plasmas is the Low confinement mode to High confinement mode (L-H) transition, which is intensely studied for more than thirty years since it provides a confinement improvement necessary for the realization of the fusion reactor. An essential issue in the L-H transition physics is the mechanism of the abrupt “radial” electric field generation in toroidal plasmas. To date, several models for the L-H transition have been proposed but the systematic experimental validation is still challenging. Here we report the systematic and quantitative model validations of the radial electric field excitation mechanism for the first time, using a data set of the turbulence and the radial electric field having a high spatiotemporal resolution. Examining time derivative of Poisson’s equation, the sum of the loss-cone loss current and the neoclassical bulk viscosity current is found to behave as the experimentally observed radial current that excites the radial electric field within a few factors of magnitude.

Self-organization in the structure-turbulence system has been widely recognized as one of the central issues in modern physics. An abrupt reduction of the turbulent transport in toroidal plasmas, i.e., the L-H transition^1^, is the prototypical example of the turbulence structure formation in high temperature plasmas, and has been intensively studied. In recent decades, much progress has been achieved in understanding of the physical mechanism, such as proposal of theoretical models for the electric field bifurcation^2^,^3^,^4^ and the turbulence suppression^5^,^6^, experimental confirmation of the radial electric field^7^,^8^,^9^, and others. Time derivative of Poisson’s equation is examined to elucidate responsible physics of the electric field bifurcation^10^, and has been applied to the plasmas in the Compact Helical System^11^. Electrode biasing experiment^12^ has provided a way to understand the electric field bifurcation and the structure formation^13^,^14^,^15^. Consecutive emergence and decay of the transport barrier just before the L-H transition, the so-called limit-cycle oscillation (LCO), are known to occur spontaneously, above which the physics of the H-mode has been discussed intensively (see refs 11,16, 17, 18 and references therein). The important role of the enhancement of the toroidal dielectric constant^10^ has been known^19^, including an experimental confirmation of the toroidal return flow^20^,^21^. Today, the quantitative validation of physical processes that include the toroidal effects of the dielectric constant can be investigated.

Here we perform systematic and quantitative validation study for the key physics for the radial electric field excitation in the L-H transition. The experiments were conducted in the JFT-2M tokamak. Heavy Ion Beam Probe (HIBP) measurement was performed for direct observations of the radial electric field, the density gradient length, and the turbulent electrostatic potential fluctuation with a high spatiotemporal resolution. Taking into account the toroidal effect on the dielectric constant, we found that the sum of the loss-cone loss current and the neoclassical bulk viscosity current^10^ meets the experimentally observed current that excites the radial electric field during the L-H transition. A small contribution of the turbulent Reynolds stress was also confirmed. This is the first experimental study that quantitatively and systematically validates the theoretical models of the electric field bifurcation in the L-H transition.

## Results

### Experimental setup

It is known that the L-H transition occurs with a heating power above a threshold. The experiments were conducted with the marginal condition (slightly above the threshold heating power) for the L-H transition. See “Methods” for more details of the plasma parameters. Multipoint measurement of the electrostatic potential *ϕ* and the electron density *n*_e_ is performed with a heavy ion beam probe (HIBP). In the series of experiments, the radial positions of the HIBP sample volumes were scanned in the edge region(−5 cm < *r* − *a* < 0 cm, where *r* − *a* is the radial distance from the saparatrix), in a shot-to-shot basis. The two key parameters of the study, the radial electric field *E*_*r*_ ≡ −∂*ϕ*/∂*r* and the inverse density gradient length 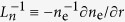, are evaluated by taking the difference of two HIBP signals measured at neighboring sample volumes. The advantage of the HIBP measurement is that one can directly obtain the radial electric field even during the transition, unlike some indirect methods that employ the radial force balance equation.

### L-H transition in the discharge

**Figure 1:**
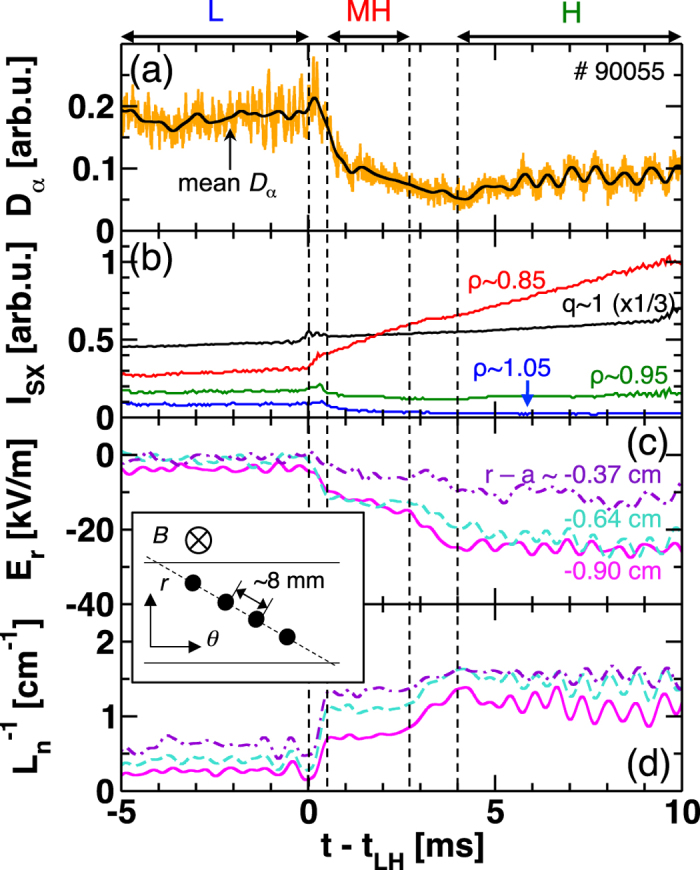
Time traces of (**a**) *D*_*α*_ emission, (**b**) soft-x-ray emission intensity *I*_SX_, (**c**) radial electric field *E*_*r*_, and (**d**) inverse density gradient length 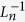 at *r* − *a* ~ −0.6 ± 0.3 cm. Radial position of *E*_*r*_ and 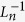 signals are labeled in Fig. 1(c) in color. The insert shows a schematic view of the measurement configuration, where two horizontal lines and four circles show the magnetic surfaces and the center of HIBP measurement positions, respectively.

**Figure 2:**
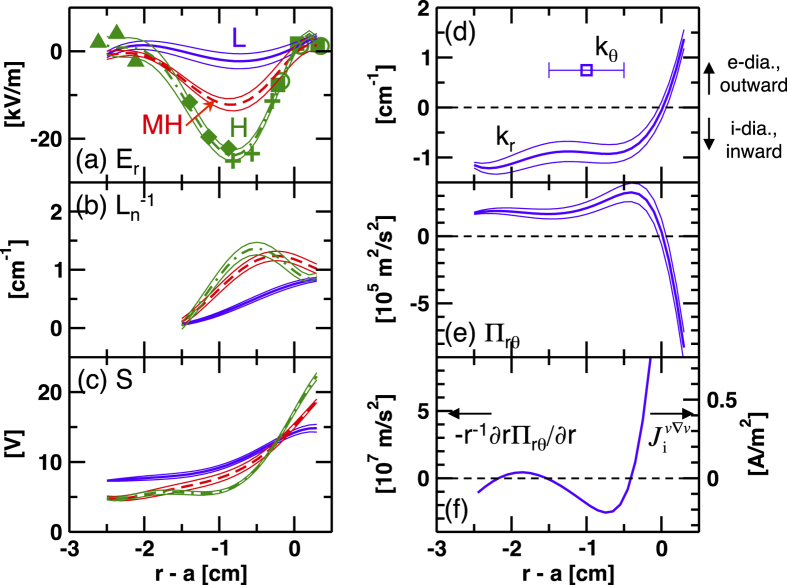
Mean radial profiles of (**a**) radial electric field *E*_*r*_, (**b**) inverse density gradient length 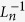, (**c**) turbulence amplitude *S*, (**d**) radial and poloidal turbulence wavenumbers *k*_*r*_ (curves) and *k*_*θ*_ (open squares), respectively, (**e**) turbulent Reynolds stress Π_*rθ*_, and (**f**) negative divergence of turbulent Reynolds stress −*r*^−1^∂*r*Π_*rθ*_/∂*r*.

Figure 1 shows the typical time evolution of (a) the edge *D*_*α*_ emission, (b) the soft-x-ray emission intensity *I*_SX_, (c) mean value of the radial electric field *E*_*r*_, and (d) mean value of the inverse density gradient length 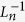 at a discharge (shot number #90055). The insert shows a schematic view of the measurement configuration. The measurement position is *r* − *a* ~ −0.6 ± 0.3 cm, where the bottom of the electric field well appears in the H-mode [see Fig. 2(a) for the *E*_*r*_ profile.]. Here the poloidal direction *θ* is taken in the electron-diamagnetic drift direction following the right-hand rule (*r, θ, z*), where the toroidal magnetic field direction is taken as *z* direction. Mean component of the parameters is calculated by use of a digital low-pass filter with a cut-off frequency of 2 kHz, to filter out the LCO (*f* ~ 4.5 kHz) or other dynamics having higher frequencies. The discharge shows the two-step transition^22^. The onset time of the first transition is generally defined as the moment when the negative electric field starts to evolve (potential to decrease) at the position of the transport barrier. This is well correlated to the moment when the heat pulse of the sawtooth crash reaches the edge *ρ* ~ 0.95 and the edge *D*_*α*_ emission drops. We call the moment *t*_LH_, which can be defined through *I*_SX_. After a short stagnation at a meta-stable state for ~2 ms, which we call the meta-H (MH) mode, the second transition occurs. The time scale of the first growth of the radial electric field (L-MH transition) [O(100 *μ*s)] is much faster than that of the second growth (MH-H transition) [O(1 ms)]. Time trace of the soft-x-ray signal shows that the sawtooth crash and/or coinciding events might trigger the L-MH transition.

Mean profiles of the radial electric field *E*_*r*_ and the inverse density gradient length 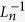 are shown in Fig. 2(a,b). Due to the restriction of the measurement volume locations, the inverse density gradient length can be computed only in the very edge region. Looking at the time evolution of the *D*_*α*_ signal in detail, five discharges with a high reproducibility and different HIBP measurement positions are selected for the use of the profile evaluation^23^. Time average is taken for each confinement state, as −5 ms ≤ *t* − *t*_LH_ ≤ 0 ms (L-mode), 1 ms ≤ *t* − *t*_LH_ ≤ 2 ms (MH-mode), and 6 ms ≤ *t* − *t*_LH_ ≤ 10 ms (H-mode). In Fig. 2(a), five kinds of symbols are plotted, showing the mean values from different discharges. A fifth-degree polynomial fits the points, where the fitting error are evaluated from the scatter of the points from the fitted curve. Through the two steps of transitions, the electric field well and its shear grow gradually. At the L-MH transition, the edge density pedestal is mostly completed, then the peak position is slightly shifted inward after the MH-H transition. The radial profile of the turbulence amplitude *S* is shown in Fig. 2(c), where it is defined as the potential fluctuation having a frequency range of 30 kHz ≤ *f* ≤ 90 kHz. The turbulence amplitude is suppressed step by step in *r* − *a* ≤ −0.3 cm. Meanwhile, it seems to grow at the outer radii. Turbulence wavenumber can also be investigated from the phase difference of the HIBP signals in the L-mode. Figure 2(d) shows the radial wavenumber *k*_*r*_ and the poloidal wave number *k*_*θ*_ in the L-mode. The horizontal error bar in the open square shows an uncertainty of the measurement position for the *k*_*θ*_ determination. Considering the Doppler shift by a small *E* × *B* velocity in the L-mode, the turbulence is found to propagate in the electron-diamagnetic drift direction in both the laboratory frame and the plasma frame. The density and potential turbulent fluctuations have almost the same relative amplitudes, a high cross coherence, and the phase difference of (0.1 − 0.2) × 2*π* (drift waves linearly unstable)^18^. Therefore, the instability is categorized to drift waves.

### Model validation

Bifurcation of the radial electric field is discussed through examining the ambipolar condition of the radial current^2^,^3^. We start from time derivative of Poisson’s equation


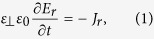


where *ε*_⊥_ is the relative dielectric constant of toroidal plasmas^10^,^19^. It is given as


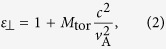


where *c*/*v*A denotes the ratio between the speed of light and the Alfvén velocity 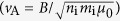. Inertia enhancement factor in the banana regime is given as


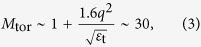


where *q* is the safety factor and *ε*_t_ = *a*/*R* is the inverse aspect ratio. In the toroidal devices, the inertia enhancement factor takes a larger value than unity. Since the *E* × *B* velocity in the poloidal direction has a finite divergence due to the toroidal geometry, the return flow that maintains the divergence free condition is generated in the direction of the magnetic field line. As a result, effective acceleration in the poloidal direction is reduced by the factor *M*_for_^10^,^19^. The toroidal return flow is experientially observed in ASDEX-U^20^ and JT-60U^21^.

The model of the radial current is given as


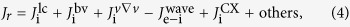


where the terms in the r.h.s. are contributions of the loss-cone loss, the neoclassical bulk viscosity, the Reynolds stress, the wave convection, and the charge exchange^10^. In ref. 24, quasi-linear part of the wave convection term 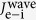 is related to quasi-linear contribution in the Reynolds stress term 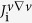 for the case of drift waves. In this discharge, the carbon wall and the carbon divertor seem to make the neutral reflection on the wall relatively weak, which results in a low neutral density in the confinement region^25^. Therefore, we do not take into account 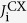. The first three terms can be quantitatively examined here at the location of the transport barrier, *r* − *a* ~ −0.6 cm. For the fourth term 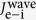, only an intuitive model is available^2^, which is considered to be important in the L-mode and will be discussed at the later part.

**Figure 3:**
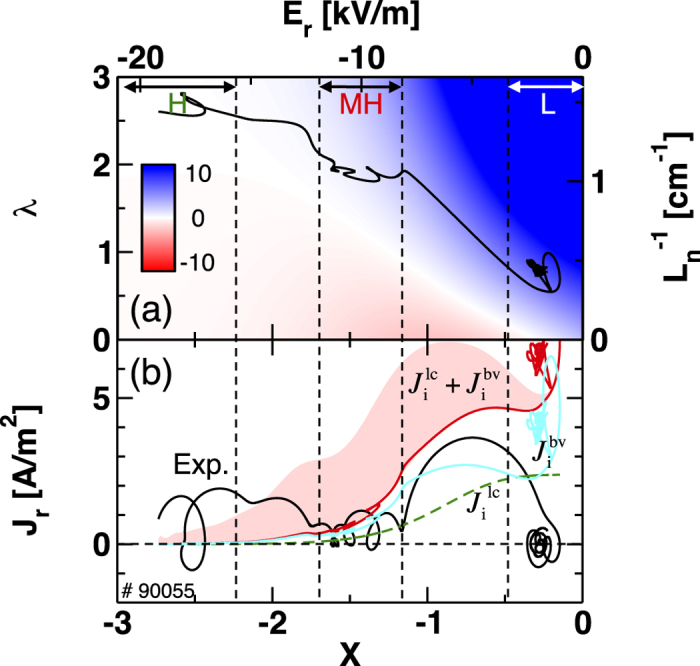
(**a**) Theoretical prediction of net radial current density (the sum of the loss-cone loss current 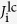 and the neoclassical bulk viscosity current 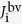) as a function of normalized radial electric field *X* and normalized inverse density gradient length *λ*. The trajectory of the experimental parameters (*X, λ*) is overplotted. (**b**) Radial current densities (experimental observation, total theoretical prediction, and each term 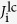 and 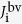) as a function of normalized radial electric field. The trajectories for 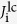 and 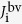 are for the case assuming ∂*T*_i_/∂*t* = 0.

First, the loss-cone loss current 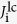 and the neoclassical bulk viscosity current 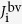 are discussed. These are given as a function of the normalized radial electric field, *X* ≡ *ρ*_p_*eE*_*r*_/*T* (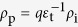 is the ion gyroradius at the poloidal magnetic field and *ρ*_i_ is the ion gyroradius), and the normalized inverse density gradient length, 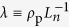, as,


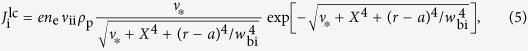


and


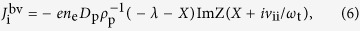


where ν_*_ = *ν*_ii_/*ω*_t_*ε*^3/2^ is the ion collisionality defined as a ratio of the ion-ion collision frequency *ν*_ii_ and the ion transit angular frequency *ω*_t_, 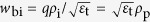 is the banana width, and 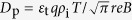 is a typical diffusivity. Plasma dispersion function Im Z(*X* + *iv*_ii_/*ω*_t_) is given in ref. 26 as a similar function of Gaussian function exp(−*X*^2^). The loss-cone loss current 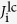 is caused by the nonambipolar particle flux due to the direct ion loss at the plasma boundary^2^. The neoclassical bulk viscosity current 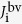 includes the *diamagnetic term* in the driving force of the radial electric field^3^,^13^, which is now thought to play a leading role in the L-H transition in high collisionality plasmas^27^. The term *λ* in the expression of 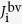 [Eq. (6)] indicates the density gradient contribution for the radial electric field excitation. Theoretical prediction of the sum of the two terms 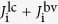 is given in Fig. 3(a), as a function of the normalized radial electric field *X* and the normalized inverse density gradient length *λ*. As a first step, we used the plasma parameters such as *ν*_ii_ and *ρ*_p_ in the L-mode for the evaluation of the models although they vary during the transition. The trajectory of the experimental parameters (*X, λ*) is overplotted. The predicted radial current is the value of the contour on the trajectory. Figure 3(b) shows the experimentally observed radial current density *J*_*r*_ = −*ε*_⊥_*ε*_0_∂*E*_*r*_/∂*t* [Eq. (1)] and the theoretical prediction 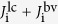 [Eqs (5) and (6)] as a function of the normalized radial electric field. The evaluations of both 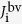 and 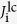 strongly depend on the value of the ion temperature. It is worthwhile to estimate uncertainty of the prediction caused by change of the ion temperature during the transition. Unfortunately, the ion temperature signal with a high time resolution is not available for the discharge. Instead, an edge electron temperature signal obtained from the electron cyclotron emission system, *T*_e_, is used for estimating the time evolution of the ion temperature *T*_i_. The shaded area in Fig. 3(b) is the uncertainty of the prediction. The upper and lower boundaries correspond to the cases *T*_i_ = *T*_e_ and ∂*T*_i_/∂*t* = 0, respectively. During the L-MH transition at *X* ~ −1, 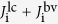 is in agreement with the observation within the factor of ~2. Contributions from 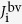 and 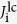 seem to be in the same order in the case ∂*T*_i_/∂*t* = 0 [shown in Fig. 3(b)], meanwhile under the case *T*_e_ = *T*_i_ the contribution of 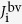 dominates that of 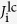. After the L-MH transition, 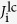 and 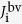 are reduced due to the large radial electric field. The peak in the *E*_*r*_ − *J*_*r*_ curve at *X* ~ −2 for the MH-H transition is not clear in these terms, since the established models do not focus on multi-step transitions. Moreover, in the L-mode, a finite positive offset of the radial current ~+5 A/m^2^ is predicted by these two terms even in the stationary state, ∂*E*_*r*_/∂*t* = 0.

Next, the contribution of the Reynolds stress term is discussed. The turbulent Reynolds stress is defined as 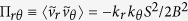. The negative divergence of the Reynolds stress, −*r*^−1^∂*r*Π_*rθ*_/∂*r*, represents the net influx of the poloidal momentum into the radius of interest, i.e., the Reynolds force. Corresponding radial current is given as


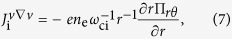


where *ω*_ci_ is the ion gyro angular frequency. Radial profile of Π_*rθ*_ and −*r*^−1^∂*r*Π_*rθ*_/∂*r* in the L-mode are shown in Fig. 2(e,f). Here we assume that the poloidal wavenumber *k*_*θ*_ to be constant within the radial turbulence correlation length of several centimeters at the edge^14^,^28^. The radial current induced by the turbulent Reynolds stress is given as 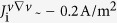 at *r* − *a* ~ −0.6 cm in the L-mode, which has only a small contribution compared with the other terms. In order to alter this result, an order of magnitude change in *k*_*θ*_ within the turbulence correlation region is required, which is not reasonable. The large positive current of 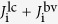 in the L-mode [Fig. 3(b)] is not compensated with the current caused by the Reynolds stress. The unimportant role of the Reynolds stress was also confirmed in the LCO in the present discharge^18^. At the plasma boundary −*r*^−1^∂*r*Π_*rθ*_/∂*r* and 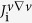 have a large positive value. The boundary limits the radial propagation of the turbulence which brings a flip of the sign in the radial wavenumber and therefore the large gradient in Π_*rθ*_. The current balance in the plasma edge should also be discussed in future. In the MH-mode, the turbulent Reynolds stress and Reynolds force decay down below one half of the values in the L-mode, and almost disappear after the final MH-H transition.

## Discussion and Summary

In the L-mode, the sum of the first three terms in Eq. (4), 
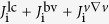
, does not agree with the experimental observation, which indicates importance of the other contributions. The most probable candidate that can achieve the ambipolar condition in the L-mode is the wave convection current term 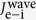, which is related to the convective loss of the wave momentum. An intuitive model of 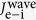 is given in ref. 2 as


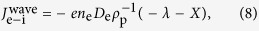


where *D*_e_ is the typical turbulent diffusivity and should be a function of *E*_*r*_. In the present case 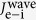 leads to a negative current whose absolute value is comparable with that of 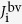 when 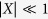, i.e., in the L-mode. This can compensate the excess prediction by 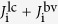 in the L-mode. After the L-H transition where the turbulence activity is suppressed, impact of 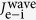 may sharply decrease as *E*_*r*_ grows. This qualitatively explains the fact that the larger *E*_*r*_ grows, the smaller the deviation between the experiential *J*_*r*_ and the model prediction 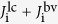 becomes as shown in Fig. 3(b). More detailed modeling for 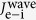 term is needed to improve quality of the prediction. Furthermore, this term is considered to be important not only for the prediction of the radial current but also for clarifying the thermal turbulent transport^29^,^30^.

In summary, the following conclusions can be made. The discharge is characterized by a two-step transition, i.e., the L-meta-H (MH) transition and the MH-H transitions. Examining time derivative of Poisson’s equation, it is found that the sum of the loss-cone loss current and the neoclassical bulk viscosity current behaves similar to the experimentally observed radial current within a few factors of magnitude during the L-MH transition. The Reynolds stress term only plays a minor role. The MH-H transition cannot be explained with the present models. In the L-mode, the sum of the loss-cone loss current and the neoclassical bulk viscosity current provides an excess positive current offset, indicating importance of the other terms. The wave convection current might be a candidate to satisfy the ambipolar current condition in the L-mode, but further modeling works are needed for more quantitative conclusion.

## Methods

### JFT-2M

JFT-2M is a medium size tokamak with a major radius (*R*) of 1.3 m and an averaged minor radius (*a*) of 0.3 m. The present experimental conditions are as follows; the neutral beam injection (NBI) power *P*_NB_ of 750 kW, the toroidal magnetic field *B* of 1.17 or 1.28 T, the safety factor at the flux surface enclosing 95% of the total poloidal flux, *q*_95_, of 2.9, the plasma current *I*_p_ of 190 kA, and the line averaged electron density 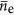 of 1.1 × 10^19^ m^−3^ before the L-H transition. At the plasma edge, the ion collisionality is slightly below unity, so that the neoclassical transport is in the banana regime. An upper single-null divertor configuration is employed, where the 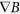 drift of ions is directed toward the X-point. JFT-2M has been shutdown in 2004.

### Heavy Ion Beam Probe (HIBP)

The HIBP on JFT-2M has a primary beam energy of *W*_0_ = 350 keV. The electrostatic potential *ϕ* and the electron density *n*_e_ can be simultaneously measured at four sample volumes (6 mm × 2 mm) on the different magnetic surfaces^31^. Radial distance between each sample volume projected in the outer midplane is ~2.5 mm. Sampling time of the system is 1 *μ*s, so that structure and turbulence can be measured simultaneously. Precise tuning of the HIBP measurement conditions, such as the primary beam energy, the toroidal magnetic field, and the incident angle of the beam, realizes two different measurement setups: the angle between the row of the sample volumes and the normal vector of the magnetic surface is set to be either 0 or ~*π*/3^31^. The poloidal wavenumber of turbulence *k*_*θ*_ can be determined by the former setup. The radial wavenumber *k*_*r*_ can be deduced from the poloidal wavenumber *k*_*θ*_ and the wavenumber along the line of the sample volumes *k*_path_, which is evaluated from the latter condition^18^. The latter condition can also be used for the radial profile measurement, assuming that the plasma parameters such as the electrostatic potential and the electron density are constant on a magnetic surface.

## Additional Information

**How to cite this article**: Kobayashi, T. *et al*. Experimental Identification of Electric Field Excitation Mechanisms in a Structural Transition of Tokamak Plasmas. *Sci. Rep.* **6**, 30720; doi: 10.1038/srep30720 (2016).
